# Management of Pain in the Intensive Care Unit: A Nordic Survey

**DOI:** 10.1111/aas.70305

**Published:** 2026-07-21

**Authors:** Benedikte Kollerup Madsen, Morten Hylander Møller, Stine Estrup, Lone Musaeus Poulsen, Bodil Steen Rasmussen, Kirstine Sylvester Conradsen, Thomas Lass Klitgaard, Asger Granfeldt, Theis Skovsgaard Itenov, Hans Christian Thorsen Meyer, Signe Tellerup Nielsen, Susanne Andi Iversen, Klaus Tjelle Kristiansen, Morten Heiberg Bestle, Kirsten Møller, Peter Hasse Møller Sørensen, Jeppe Veien Nygaard, Kristian Elgaard, Morten Rune Blichfeldt‐Eckhardt, Ann Christine Waarkjær Olsen, Mette Pedersen, Henrik Westy Hoffmeyer, Peter Martin Hansen, Helle Bundgaard, Marcus Ølgaard Møller, Jarl Sigaard, Kim Zillo Rokamp, Anne Craveiro Brøchner, Christoffer Sølling, Mika Valtonen, Anna‐Maria Kuivalainen, Annukka Vahtera, Stepani Bendel, Martin I. Sigurdsson, Per Martin Bådstøløkken, Fredrik Sjövall, Ole Mathiesen, Lars Peter Kloster Andersen

**Affiliations:** ^1^ Department of Anesthesiology Centre for Anesthesiologic Research, Zealand University Hospital Køge Denmark; ^2^ Department of Intensive Care Centre for Cancer and Organ Diseases, Copenhagen University Hospital—Rigshospitalet Copenhagen Denmark; ^3^ Department of Clinical Medicine, Faculty of Health Sciences University of Copenhagen Copenhagen Denmark; ^4^ Department of Clinical Medicine Aalborg University Aalborg Denmark; ^5^ Department of Anesthesiology and Intensive Care Aalborg University Hospital Aalborg Denmark; ^6^ Section for Neuro and Trauma Intensive Care (NOTIA) Aalborg University Hospital Aalborg Denmark; ^7^ Section for Cardio‐Thoracic and Vascular Anesthesia and Intensive Care Aalborg University Hospital Aalborg Denmark; ^8^ Department of Anesthesiology and Intensive Care Aarhus University Hospital Aarhus Denmark; ^9^ Department of Anesthesiology and Intensive Care Copenhagen University Hospital—Bispebjerg Hospital Copenhagen Denmark; ^10^ Department of Anesthesiology and Intensive Care Copenhagen University Hospital—Bornholm Hospital Bornholm Denmark; ^11^ Department of Anesthesiology and Intensive Care Copenhagen University Hospital—Herlev Hospital Herlev Denmark; ^12^ Department of Anaesthesiology and Intensive Care Slagelse Hospital Slagelse Denmark; ^13^ Department of Anesthesiology and Intensive Care Copenhagen University Hospital—Hvidovre Hospital Hvidovre Denmark; ^14^ Department of Anesthesiology and Intensive Care Copenhagen University Hospital—North Zealand Hospital Hilleroed Denmark; ^15^ Department of Neuroanesthesiology Copenhagen University Hospital—Rigshospitalet Copenhagen Denmark; ^16^ Department of Cardiothoracic Anesthesiology (4141) Copenhagen University Hospital—Rigshospitalet Copenhagen Denmark; ^17^ Department of Anesthesiology and Intensive Care Goedstrup Hospital Goedstrup Denmark; ^18^ Department of Anesthesiology and Intensive Care Holbaek Hospital Holbaek Denmark; ^19^ Department of Anesthesiology and Intensive Care Lillebaelt Hospital Vejle Denmark; ^20^ Department of Anesthesiology and Intensive Care North Denmark Regional Hospital Hjoerring Denmark; ^21^ Department of Anesthesiology and Intensive Care Odense University Hospital Odense Denmark; ^22^ Department of Neurosurgical Intensive Care (NIA) Odense University Hospital Odense Denmark; ^23^ Department of Anesthesiology and Intensive Care Odense University Hospital Svendborg Denmark; ^24^ Department of Anaesthesiology and Intensive Care Randers Regional Hospital Randers Denmark; ^25^ Department of Anesthesiology and Intensive Care University Hospital of Southern Denmark, Aabenraa Hospital Aabenraa Denmark; ^26^ Department of Anesthesiology and Intensive Care University Hospital of Southern Denmark Esbjerg Denmark; ^27^ Department of Anaesthesiology and Intensive Care Zealand University Hospital Nykoebing Falster Denmark; ^28^ Department of Anaesthesiology and Intensive Care University Hospital of Southern Denmark Kolding Denmark; ^29^ Department of Anesthesiology and Intensive Care Viborg Regional Hospital Viborg Denmark; ^30^ Intensive Care Unit Turku University Hospital, Wellbeing Services County of Southwest Finland Turku Finland; ^31^ Intensive Care Unit Helsinki University Hospital Helsinki Finland; ^32^ Intensive Care Unit Tampere University Hospital, Wellbeing Services County of Pirkanmaa Tampere Finland; ^33^ Intensive Care Unit Kuopio University Hospital, Wellbeing Services County of North Savo Kuopio Finland; ^34^ Department of Anesthesiology and Intensive Care Medicine Landspital—The National University Hospital of Iceland Reykjavik Iceland; ^35^ Department of Intensive Care Akershus Universitetssykehus Oslo Norway; ^36^ Department of Intensive Care Skaane University Hospital Malmoe Sweden

## Abstract

**Background:**

Pain is common in intensive care unit (ICU) patients with up to one‐third experiencing pain at rest and even more during mobilization and clinical procedures. This survey aimed to explore physicians' attitudes and preferences regarding pain management in adult ICU patients in the Nordic countries.

**Methods:**

We conducted an electronic survey targeting physicians working regularly in an ICU in the Nordic countries: Denmark, Finland, Iceland, Norway and Sweden. The survey focused on pain assessment, pharmacological treatments, and post‐discharge follow‐up of adult ICU patients.

**Results:**

The survey was distributed to 606 physicians, and 360 responses were received (overall response rate 59%). Respondents were primarily from Denmark, with only a few respondents from the remaining Nordic countries. Most respondents were specialists in anesthesiology working in mixed ICUs in public specialist hospitals. Standardized pain assessment tools were widely used in non‐sedated patients, while only half of respondents employed standardized pain assessment tools in sedated patients. Respondents reported that pain was assessed multiple times daily in both non‐sedated and sedated patients. Daily wake‐up calls in sedated patients were considered important by almost all respondents. Morphine was the preferred opioid for oral‐ and intravenous bolus administration, while remifentanil was preferred for intravenous continuous administration. However, preferences varied across countries with regard to both opioid‐ and non‐opioid analgesics. Nearly half of respondents expressed concerns regarding the development of opioid‐induced hyperalgesia. Methadone was the most frequently preferred drug for opioid weaning, although preferences varied between countries. Most respondents acknowledged the importance of ICU‐follow up programs, but only about half of respondents reported that their ICU currently offered a follow‐up service.

**Conclusion:**

This Nordic survey explored ICU physicians' attitudes and preferences regarding pain management in adult ICU patients. Respondents reported assessing pain frequently, employing standardized pain assessment tools primarily in non‐sedated patients. Daily wake‐up calls in sedated patients were generally perceived as important. Interestingly, preferences regarding opioid‐ and non‐opioid analgesics varied between Nordic countries. ICU‐follow up programs were recognized as important but were not consistently implemented. Despite a high overall response rate, the generalizability of our findings is impaired by limited participation in most Nordic countries except Denmark.

**Editorial Comment:**

This survey describes the physician perspective on predominantly pharmacological pain management in selected ICUs within the Nordic countries.

## 1 Introduction

Pain is common among patients in the intensive care unit (ICU) [1]. Studies demonstrate that up to one third of patients experience pain at rest, with an even higher proportion experiencing pain during diagnostic or therapeutic procedures [2, 3, 4]. Pain is associated with increased risk of delirium, prolonged duration of mechanical ventilation and ICU stay, and may contribute to the development of post intensive care syndrome (PICS) and chronic pain states [2, 5, 6, 7].

Despite the substantial impact on patient experience and outcomes, previous research documents considerable variation in pain management in ICU patients [8, 9, 10, 11, 12]. Furthermore, analgesic treatment strategies evolve over time as new analgesics and treatment approaches are introduced in the clinic. Hence, further investigations are needed to characterize ICU physicians' contemporary pain management practices.

In this survey, we aimed to explore physicians' attitudes and preferences regarding pain management treated in adult ICU patients in the Nordic countries.

## 2 Methods

### 2.1 Survey Design and Approvals

We conducted a cross‐sectional online survey targeting physicians (specialists and non‐specialists) working regularly in ICUs in either Denmark, Finland, Iceland, Norway, or Sweden. We employed a secure web application (Research Electronic Data Capture; REDCap) hosted by Region Zealand, Denmark [13, 14]. The survey was approved by the Legal Department of Scientific Research in Region Zealand (approval number: REG‐066‐2023). In coherence with Danish law, other approvals were waived as no patient data were collected and work‐emails provided by respondents were voluntary. The manuscript was prepared in accordance with the Consensus‐Based Checklist for Reporting of Survey Studies (CROSS) checklist (Supplement S1).

### 2.2 Survey Content and Completion

The survey consisted of 26 mandatory questions in English designed by the managing committee (B.K.M., L.P.K.A., B.S.R., O.M., L.M.P., S.E., M.H.M.). In two questions, an additional multiple‐choice question was generated if respondents selected the “yes” option, and in seven questions, branching logic was employed to display an open‐ended text field when respondents selected the ‘other’ option. This allowed respondents to elaborate their response if none of the predefined options accurately reflected their preferences/values/attitudes. Four questions related to the respondents' demographics. Five questions addressed preferences regarding pain assessment practices. Eight questions examined values in relation to sedation practices and preferred pharmacological treatments. Six questions explored respondents' values in relation to opioid‐induced hyperalgesia, opioid tolerance and opioid weaning. Three questions investigated respondents' preferences regarding ICU follow‐up services. Finally, the respondents were asked to provide their work‐email address to avoid duplicate responses. Completion of the survey was considered as informed consent. The survey was designed to be completed in approximately 10 min. The survey was pilot tested for content validity, clarity of questions, and completion time by 10 clinicians before survey initiation. This process resulted in minor adjustments of the phrasing of the questions. The distributed survey is available in Supplement S2.

### 2.3 Survey Distribution and Data Collection

Site investigators were identified through the professional network of the managing committee and websites of the local hospitals (Supplement S3). Site investigators were responsible for evaluating which of their colleagues were within the defined target group of the survey, responsible for distributing the survey, and motivating their colleagues to answer the survey. The site investigator reported the total number of physicians who received the email link to the survey. Two reminder emails were sent if needed during the study period. The survey was distributed between the 17th of June and the 15th of September 2025. No financial compensation was provided to either site investigators or respondents. In case of duplicate responses, the most recent response was included.

### 2.4 Statistics

Data are reported descriptively as counts/percentages. Statistical analyses were performed using R (Version 4.5.1) and Microsoft Excel. Analyses are based exclusively on reported data with no data imputation applied.

## 3 Results

The survey was distributed to 606 respondents in 5 countries. Seven duplicate responses were removed. Three hundred and sixty respondents completed the survey, providing an overall response rate of 59%. Missing data was apparent in 0.6% of responses (see Supplement S4). Country‐specific response rates varied from 14% (Sweden) to 70% (Denmark). Respondents were primarily specialist physicians in anesthesiology (91%). Most respondents (77%) worked in mixed ICUs. Fifty percent of respondents worked in a public specialist hospital (Table 1).

**TABLE 1:** Characteristics of respondents.

	Number of respondents	Response rates (number of respondents/number of invited physicians)
**Country**		
Denmark	279 (77.5%)	279/396 (70.5%)
Finland	43 (11.9%)	43/93 (46.3%)
Iceland	22 (6.1%)	22/54 (40.7%)
Norway	12 (3.3%)	12/35 (34.3%)
Sweden	4 (1.1%)	4/28 (14.3%)
**Hospital type**		
Public general hospital^a^	177 (49.2%)	
Public specialist hospital^b^	183 (50.8%)	
Private general hospital^a^	0 (0.0%)	
Private specialist hospital^b^	0 (0.0%)	
**Specialist physician**		
Yes	330 (91.7%)	
No	30 (8.3%)	
**Type of ICU**		
Mixed	279 (77.5%)	
Surgical	3 (0.8%)	
Medical	0 (0.0%)	
Neuro‐intensive	43 (11.9%)	
Cardio‐thoracic	33 (9.2%)	
Other	2 (0.5%)	

^a^ General hospital was defined as a hospital or medical center which can provide licensed physicians in internal medicine, pediatrics, obstetrics, gynecology, general surgery and other supporting medical services.

^b^ Specialist hospital was defined as a hospital which can provide similar services as described for general hospitals, and in addition provide specialized care such as neurosurgery, cardiac surgery, plastic surgery or transplantation surgery.

### 3.1 Assessment of Pain

**FIGURE 1:**
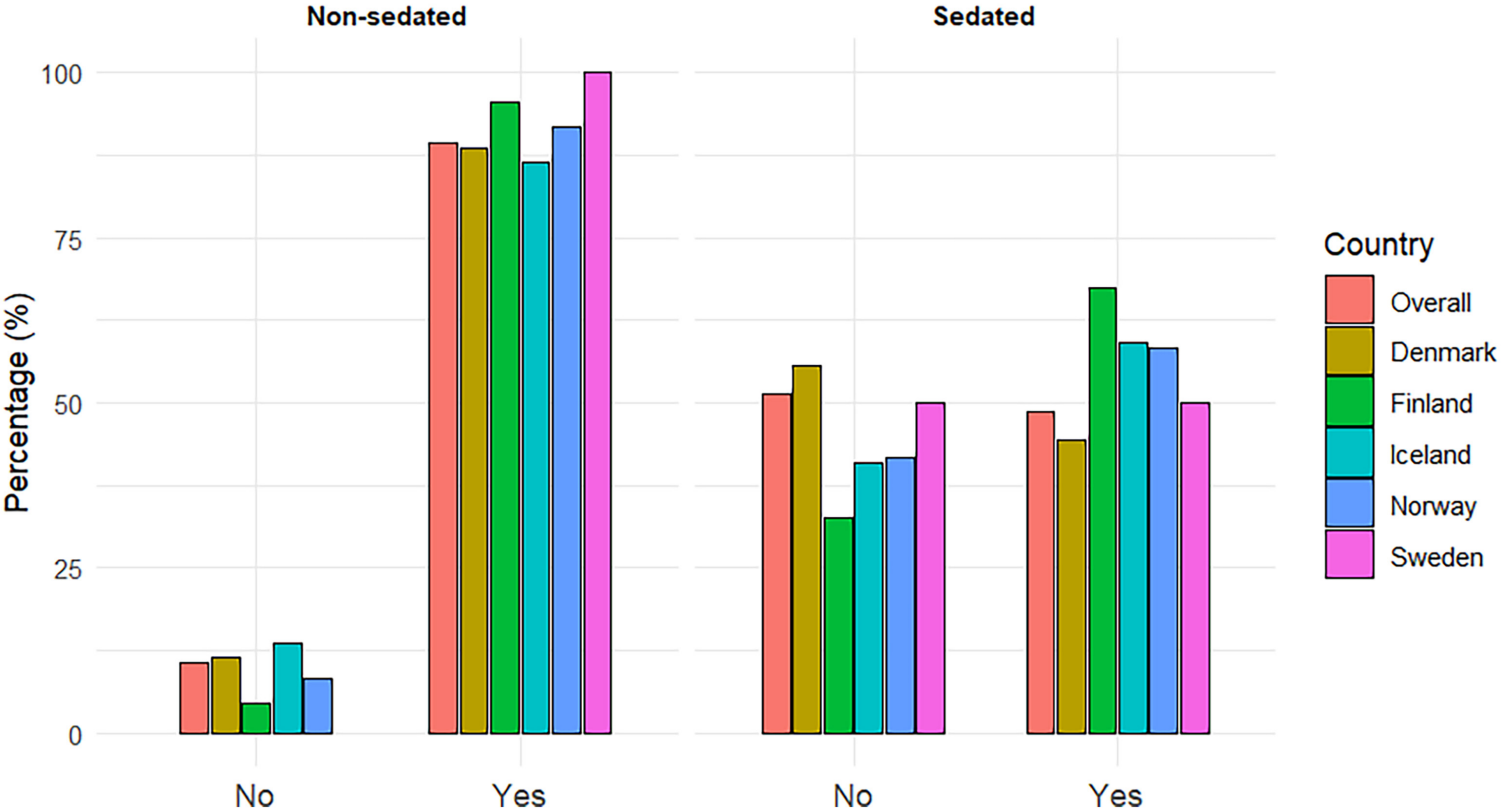
The use of standardized pain assessment tools (e.g., VAS, NRS, CPOT) in non‐sedated and in sedated ICU patients.

In non‐sedated patients, 89% of respondents preferred to use standardized pain assessment tools. In contrast, only half of respondents (49%) preferred to use standardized pain assessment tools in sedated patients (Figure 1). The Numeric Rating Scale (NRS) (51%) and The Critical Care Pain Observation Tool (CPOT) (96%) were the preferred pain assessment tools in non‐sedated and in sedated patients, respectively (Supplement S5).

Respondents reported assessing pain multiple times daily in both non‐sedated (75%) and in sedated patients (66%). Some differences across countries were observed (Supplement S6). Some respondents reported that the need for pain assessment was evaluated on an individual basis in both non‐sedated and sedated patients (18% and 21%, respectively). Respondents identified nurses as the health care professionals performing pain assessments (99%) most frequently; however, physicians (60%) and physio‐ and occupational therapists (10%) were also identified.

### 3.2 Sedation Practice and Pharmacological Treatments

Ninety‐four percent of respondents recognized daily wake‐up calls in sedated patients as important (Supplement S7). Thirty‐seven percent of respondents would almost never, 24% would occasionally, and 15% would frequently prescribe opioids as monotherapy for sedation in mechanically ventilated patients. When assessing countries individually, most respondents from Finland and Iceland (76% and 68%, respectively) reported that they would almost never prescribe opioids as monotherapy. In contrast, only 28% and 25% of Danish and Norwegian respondents, respectively, reported that they would almost never prescribe opioids as monotherapy (Supplement S8).

**FIGURE 2:**
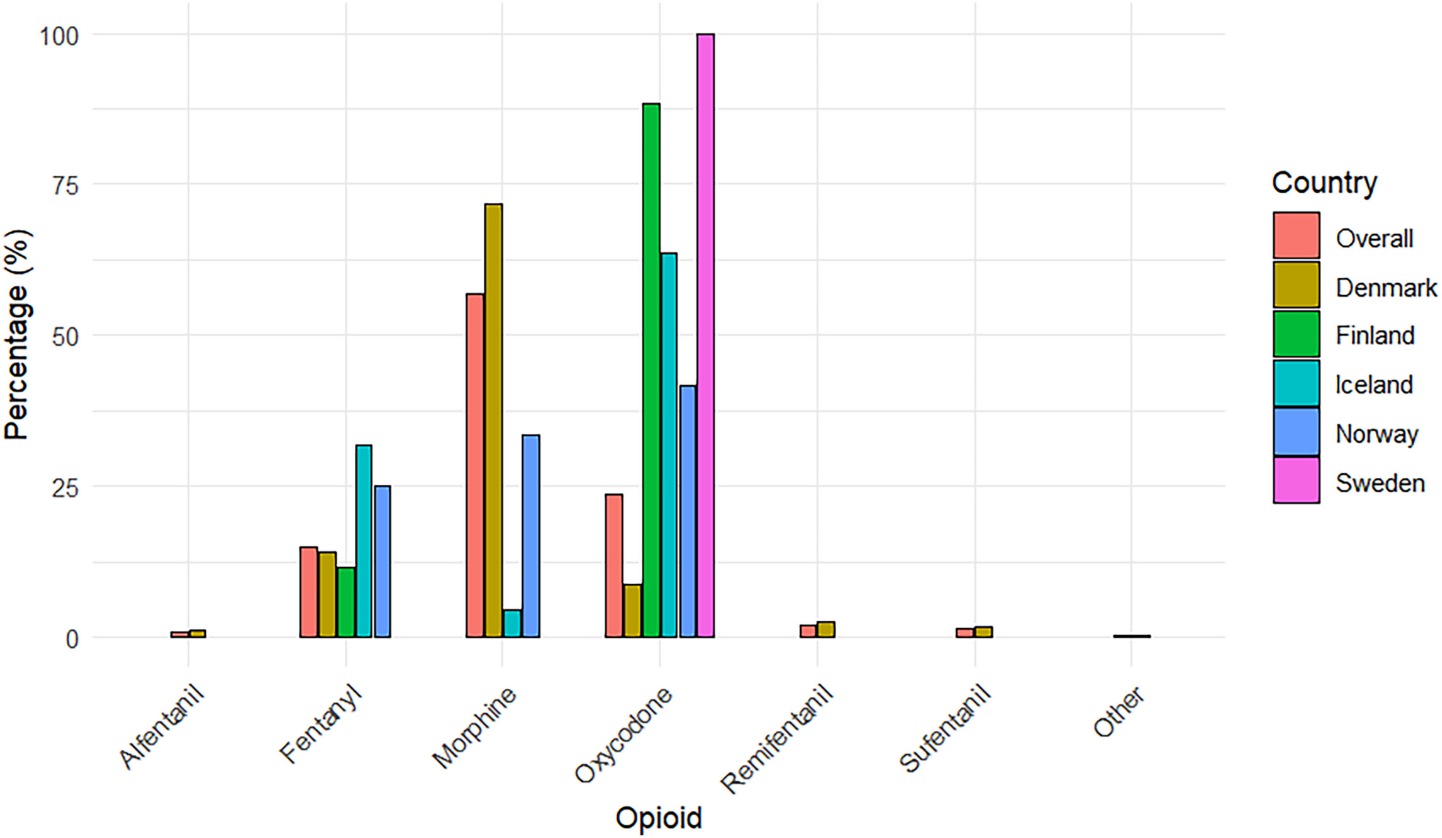
One respondent (0.3%) chose the “other” option and replied it depended on the patient and the situation.

Morphine (56%), oxycodone (23%), and fentanyl (15%) were the preferred opioids for intravenous (IV) bolus administration. When assessing countries individually, Danish respondents (71%) preferred morphine, whereas Finnish (88%), Icelandic (63%), Norwegian (41%), and Swedish (100%) respondents preferred oxycodone (Figure 2).

**FIGURE 3:**
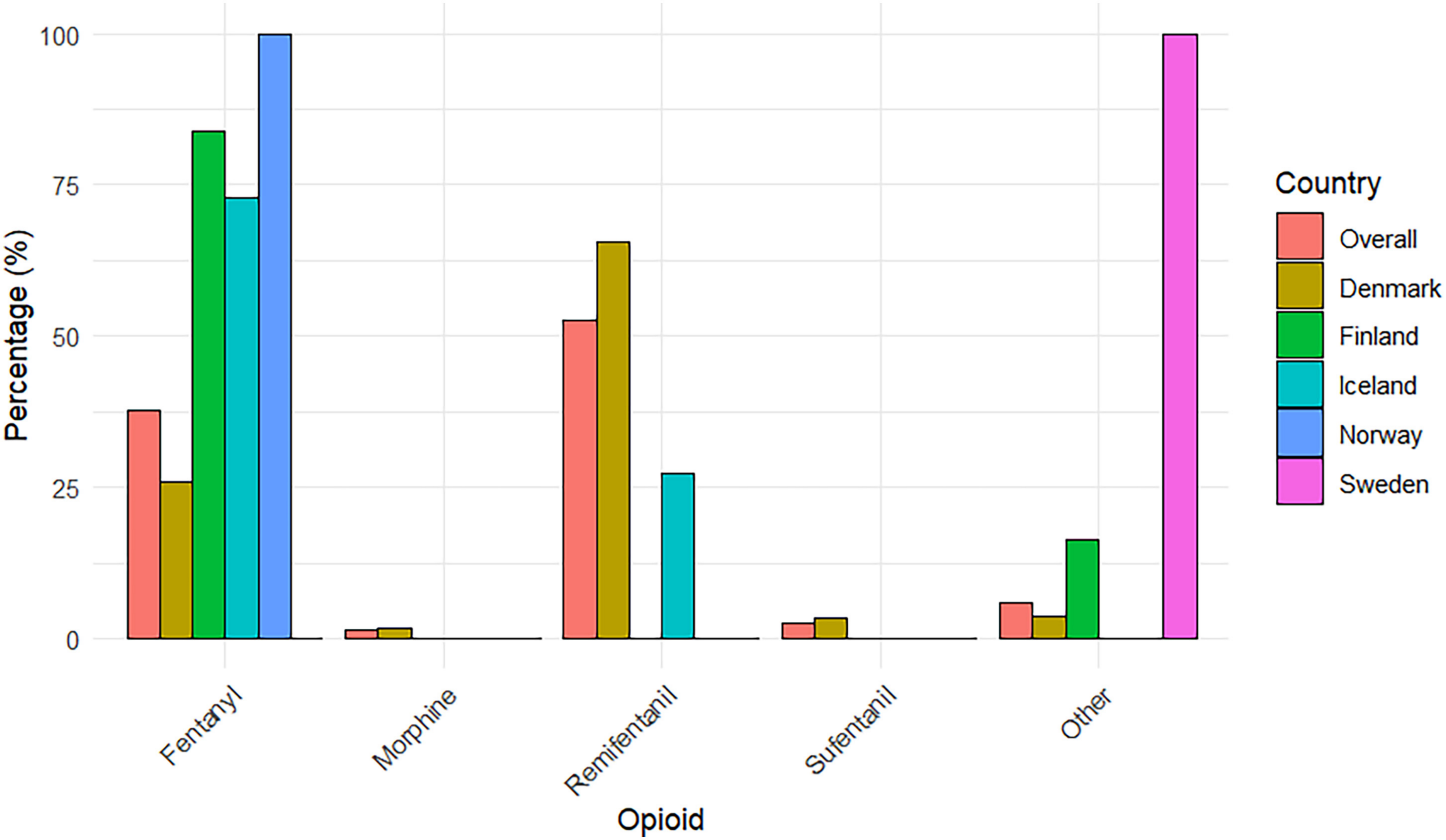
Of the 21 respondents (6%) who chose the “other” option, 10 respondents (48%) replied alfentanil, 10 respondents (48%) replied oxycodone, and 1 respondent (5%) replied it depends on the patient and the situation.

For continuous IV opioid administration, remifentanil was preferred by 53% of respondents followed by 38% preferring fentanyl. However, preferences varied across countries with Finnish, Icelandic, and Norwegian respondents preferring fentanyl (84%, 73%, and 100%, respectively), whereas Danish respondents preferred remifentanil (66%) and Swedish respondents preferred oxycodone (Figure 3).

**FIGURE 4:**
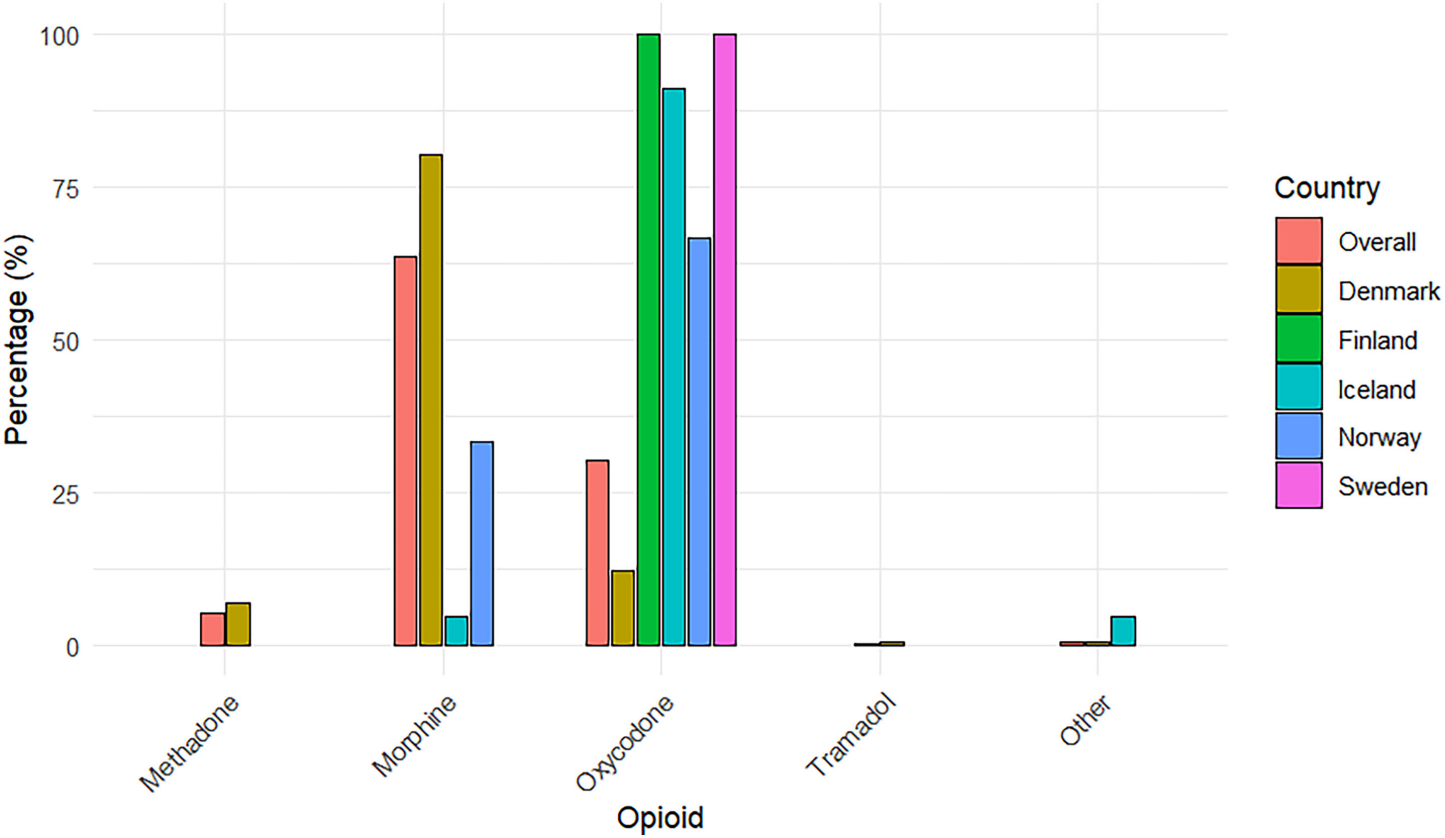
Of the 2 respondents (0, 6%) who chose the other option, 1 respondent replied oxycodone/naloxone and 1 respondent replied it depended on the patient and the situation.

For oral opioid treatment, morphine (63%) was the most preferred. When assessing countries individually, Danish respondents preferred morphine (80%), whereas respondents from Finland (100%), Iceland (90%), Norway (66%), and Sweden (100%) preferred oxycodone (Figure 4).

Paracetamol was the most‐favored non‐opioid analgesic, as 75% stated they almost always used it (Supplement S9). Respondents stated occasionally using gabapentinoids and central/peripheral nerve blockades (54%, 42% and 31%, respectively), whereas respondents stated rarely using ketamine (38%). Respondents stated almost never using nonsteroidal anti‐inflammatory drugs and tricyclic antidepressants (40% and 45%, respectively). Some differences between countries were observed (Supplement S9).

### 3.3 Opioid‐Induced Hyperalgesia and Tolerance

Forty‐seven percent of respondents reported concerns regarding patients developing opioid‐induced hyperalgesia. Country‐specific differences ranged from 41% (Iceland) to 75% (Norway and Sweden) (Supplement S10). Opioid rotation (32%) and alpha‐2 agonists (22%) were reported as preferred treatments if opioid‐induced hyperalgesia was suspected (Supplement S10); however, differences across countries were observed (Supplement S10). Three percent of respondents would almost never, 19% would rarely, 43% would occasionally, 31% would frequently, and 4% would almost always observe development of opioid tolerance (Supplement S11).

### 3.4 Weaning Plans

Respondents reported that they would occasionally (35%), frequently (28%), or almost always (13%) discharge patients with an opioid weaning plan. Seventeen and 5% of respondents would rarely or almost never prepare opioid weaning plans (Supplement S12). Methadone was the preferred drug for oral opioid weaning (41%) (Supplement S12). Differences in preferences between countries were observed (Supplement S13). Respondents reported that patients would frequently (44%) or occasionally (25%) be discharged from the ICU with a prescribed opioid. Responses were generally consistent between countries except for Finland, where a higher proportion of respondents reported almost always discharging patients with a prescribed opioid (39%) (Supplement S13).

### 3.5 ICU Follow‐Up Programs

Most respondents recognized ICU follow‐up programs as important (48%) or very important (20%) (Supplement S14). Approximately half of respondents (53%) replied that their ICU offered follow‐up programs with higher rates in Finland (93%), Iceland (81%), Norway (83%), and Sweden (100%), compared to Denmark (43%) (Supplement S14). Assessment of chronic pain in follow‐up programs was perceived as important (47%) or very important (23%) by respondents (Supplement S14).

## 4 Discussion

This Nordic survey investigated ICU physicians' attitudes and preferences in relation to pain management in adult ICU patients. Our survey found that standardized tools for pain assessment were reported to be broadly employed in the ICU, especially in non‐sedated patients. Most respondents reported that pain was assessed multiple times daily. Morphine was the preferred opioid for oral‐ and IV bolus administration while remifentanil was preferred for IV continuous administration. However, preferences varied across countries regarding both opioid‐ and non‐opioid analgesics. Most respondents reported discharging patients with an opioid weaning plan. Methadone was the preferred drug for oral opioid weaning. ICU follow‐up programs were perceived as important by most respondents, but only half of respondents replied that their ICU currently offered a follow‐up service.

Pain in the ICU may result from a variety of causes including prolonged bed rest, underlying disease, post‐surgery, trauma, mechanical ventilation, and procedures [4, 15, 16, 17, 18]. Pain is experienced by a large proportion of patients in the ICU [4, 19]. Observational studies describe that up to 63% of patients suffer from pain during their ICU admission with up to 33% of patients experiencing significant pain at rest for more than half of their ICU stay [20, 21, 22]. The significance of pain is also underlined by being one of the dominant memories reported by ICU survivors [23]. Interestingly, this is also the case in patients who were sedated during their admission [24, 25]. Pain during admission may impair convalescence by increasing the risk of delirium, the duration of mechanical ventilation, and the ICU stay [2, 5, 6, 7]. Also, pain during ICU stay may instigate the development of chronic pain [23]. Mottet et al. found that days with significant pain (defined as NRS > 3 or BPS ≥ 5) as well as opioid exposure were independent risk factors for chronic pain in ICU survivors at 3 months follow‐up [15].

In this survey, most respondents answered that they evaluated pain multiple times daily in both non‐sedated and in sedated patients. However, approximately one in five respondents would assess patients individually, which may increase the risk of inattention and possibly suboptimal analgesic treatment. Furthermore, almost half of respondents would not employ standardized tools when assessing pain in sedated patients, which highlights a possible lack of standardization in this vulnerable patient group. Our findings are in coherence with previous studies demonstrating that clinical pain management practice may differ significantly from recommendations [8]. The Society of Critical Care Medicine recommends employing a protocol‐based, stepwise approach including standardized clinical pain assessments to assess and treat pain [26]. Studies have demonstrated that standardized pain assessment in the ICU reduces the use of opioids/sedatives and reduces the duration of mechanical ventilation and ICU stay [5, 6, 27].

A previous Danish survey study from 2006 found that fentanyl and sufentanil were preferred opioids for IV continuous administration in sedated patients in Danish ICUs [11]. We found that remifentanil was now the most preferred among Danish respondents; however, respondents from Finland, Iceland, and Norway continue to prefer fentanyl. Hence, despite geographic proximity, important clinical differences exist.

Remifentanil is often employed for sedation due to the favorable pharmacokinetic profile with a short half‐life [28], however, its use has been linked to increased risk of opioid‐induced hyperalgesia [29]. Also, extensive and prolonged use of opioids in general may lead to tachyphylaxis and withdrawal symptoms [30, 31, 32]. Finally, it has been suggested that the use of opioids in the ICU may induce chronic pain states following ICU discharge [33]. Half of respondents raised concerns about opioid‐induced hyperalgesia, and over half of respondents experienced patients with suspected opioid tolerance either occasionally or often. These issues underline the pivotal importance of aiming to minimize the use of opioids in the ICU [34].

One way of sparing opioids is by employing multimodal analgesic regimes which may include paracetamol, NSAIDs, nerve blockades, and secondary analgesics. Almost all respondents prescribed paracetamol for pain. In contrast, NSAIDs, ketamine, and tricyclic antidepressants were almost never or rarely employed. Studies have demonstrated that a multimodal analgesic approach with protocolized pain assessment may reduce the use of opioids without compromising patient comfort [27, 35, 36]. One study even indicated that implementing a multimodal pain protocol reduced both morbidity and mortality in ICU patients [35]. Conclusively, when selecting individual or combinations of analgesic drugs, the clinical use in ICU patients requires careful attention due to the increased risk of adverse drug effects and interactions [37].

In this survey, most respondents perceived ICU follow‐up as important, and about half of respondents reported that their ICU provided a follow‐up service which is in alignment with a previous survey by Kjer et al. [38] Some studies have indicated a positive impact on patients' quality of life and mental health when offered follow‐up after ICU discharge [39]. A Cochrane review, however, found insufficient evidence to determine whether this clinical strategy was effective in identifying and treating health needs of ICU survivors [40]. A recent consensus guideline by The Society of Critical Care Medicine recommends four tools for assessing post‐intensive care syndrome (PICS) in high‐risk ICU survivors; however, it does not provide any recommendations regarding the assessment of chronic pain [41]. Increasing evidence suggests that chronic pain is a common long‐term consequence among ICU survivors and may contribute to the physical, psychological, and cognitive impairments associated with PICS [42, 43]. Conclusively, a gap still exists regarding the needs and challenges that ICU survivors experience in the aftermath of an ICU course [44].

## 5 Strengths and Limitations

The main strengths of this survey include a low level of missing data and a high overall response rate. Extensive collaboration with site investigators from almost all Danish ICUs enabled us to achieve a national response rate exceeding 70% in Denmark. In contrast, only one site investigator was recruited in Sweden and Norway, respectively, and together with low national response rates, the external validity of data in these countries is limited. Also, it is possible that respondents with particular interest in pain management were more likely to complete the questionnaire, introducing a possible selection bias. Another important limitation is that the survey population was restricted to physicians, though ICU pain management is a multidisciplinary effort also involving nurses and other healthcare professionals. As such, the focus of this study was primarily on pharmacological approaches in relation to pain management and did not address non‐pharmacological interventions. Finally, the survey may be subject to common methodological limitations associated with questionnaire‐based research including response fatigue, recall bias, and ambiguity in multiple‐choice response options which may explain the uniform distribution of answers displayed in some questions [45]. It is possible that some respondents may have interpreted questions as referring to their ICU team, departmental practices, or institutional protocols, instead of personal preferences. These limitations should be considered when interpreting our findings as they may affect both the validity and generalizability of the results.

## 6 Conclusion

This Nordic survey explored ICU physicians' attitudes and preferences regarding pain management in adult ICU patients. The overall response rate was 59%. Respondents reported assessing pain frequently employing standardized pain assessment tools primarily in non‐sedated patients. Daily wake‐up calls in sedated patients were generally perceived as important. Interestingly, preferences regarding opioid‐ and non‐opioid analgesics varied between Nordic countries. ICU‐follow‐up programs were recognized as important but were not consistently implemented. Despite a high overall response rate, generalizability of our findings is impaired by limited participation in most Nordic countries except Denmark.

## Author Contributions

Conceptualization and study design: B.K.M., L.P.K.A., B.S.R., O.M., L.M.P., S.E., M.H.M. Data analysis: B.K.M. Writing first draft: B.K.M., L.P.K.A., B.S.R., O.M., L.M.P., S.E., M.H.M. Critical review and approval of manuscript: all authors.

## Funding

The authors have nothing to report.

## Conflicts of Interest

The authors declare no conflicts of interest.

## Supporting information


**Supplement S1:** Checklist for Reporting Of survey Studies (CROSS).
**Supplement S2:** The distributed survey.
**Supplement S3:** Participating countries, sites and investigators.
**Supplement S4:** Missingness.
**Supplement S5:** Assessment tools.
**Supplement S6:** Pain assessment frequency.
**Supplement S7:** Wake‐up calls.
**Supplement S8:** Opioids as monotherapy for sedation.
**Supplement S9:** Non‐opioid analgesics.
**Supplement S10:** Opioid‐induced hyperalgesia.
**Supplement S11:** Opioid tolerance.
**Supplement S12:** Opioid weaning.
**Supplement S13:** Opioid prescription.
**Supplement S14:** ICU follow‐up programs.

## Data Availability

The data that support the findings of this study are available from the corresponding author upon reasonable request.
