# Supplementary material for: Management of Pain in the Intensive Care Unit: A Nordic Survey
Source: Acta Anaesthesiol Scand. 2026 Jul 21;70(8):e70305. doi: 10.1111/aas.70305 (PMC13388110; doi:10.1111/aas.70305)
Supplement: Supplementary file 1 — Supplement S1: Checklist for Reporting Of survey Studies (CROSS). Supplement S2: The distributed survey. Supplement S3: Participating countries, sites and investigators. Supplement S4: Missingness. Supplement S5: Assessment tools. Supplement S6: Pain assessment frequency. Supplement S7: Wake‐up calls. Supplement S8: Opioids as monotherapy for sedation. Supplement S9: Non‐opioid analgesics. Supplement S10: Opioid‐induced hyperalgesia. Supplement S11: Opioid tolerance. Supplement S12: Opioid weaning. Supplement S13: Opioid prescription. Supplement S14: ICU follow‐up programs. [file AAS-70-0-s001.docx]

**Title**

Management of pain in the intensive care unit: a Nordic survey

**Authors**

Benedikte Kollerup Madsen, Morten Hylander Møller, Stine Estrup, Lone Musaeus Poulsen, Bodil Steen Rasmussen, Kirstine Sylvester Conradsen, Thomas Lass Klitgaard, Asger Granfeldt, Theis Skovsgaard Itenov, Hans-Christian Thorsen-Meyer, Signe Tellerup Nielsen, Susanne Andi Iversen, Klaus Tjelle Kristiansen, Morten Heiberg Bestle, Kirsten Møller, Peter Hasse Møller Sørensen, Jeppe Veien Nygaard, Kristian Elgaard, Morten Rune Blichfeldt-Eckhardt, Ann Christine Waarkjær Olsen, Mette Pedersen, Henrik Westy Hoffmeyer, Peter Martin Hansen, Helle Bundgaard, Marcus Ølgaard Møller, Jarl Sigaard, Kim Zillo Rokamp, Anne Craveiro Brøchner, Christoffer Sølling, Mika Valtonen, Anna-Maria Kuivalainen, Annukka Vahtera, Stepani Bendel, Martin I. Sigurdsson, Per Martin Bådstøløkken, Fredrik Sjövall, Ole Mathiesen, Lars Peter Kloster Andersen

Supplementary Appendix

Table of contents

[Supplement 1: Checklist for Reporting Of survey Studies (CROSS) 2](#_Toc218017581)

[Supplement 2: The distributed survey 6](#_Toc218017582)

[Supplement 3: Participating countries, sites and investigators 11](#_Toc218017583)

[Supplement 4: Missingness 13](#_Toc218017584)

[Supplement 5: Assessment tools 16](#_Toc218017585)

[Supplement 6: Pain assessment frequency 17](#_Toc218017586)

[Supplement 7: Wake-up calls 18](#_Toc218017587)

[Supplement 8: Opioids as monotherapy for sedation 19](#_Toc218017588)

[Supplement 9: Non-opioid analgesics 20](#_Toc218017589)

[Supplement 10: Opioid-induced hyperalgesia 24](#_Toc218017590)

[Supplement 11: Opioid tolerance 25](#_Toc218017591)

[Supplement 12: Opioid weaning 25](#_Toc218017592)

[Supplement 13: Opioid prescription 26](#_Toc218017593)

[Supplement 14: ICU follow-up programs 27](#_Toc218017594)

## Supplement 1: Checklist for Reporting Of survey Studies (CROSS)

| **Section/topic** | **Item** | **Item description** | **Reported on page #** |
| --- | --- | --- | --- |
| **Title and abstract** | | |  |
| Title and abstract | 1a | State the word “survey” along with a commonly used term in title or abstract to introduce the study’s design. | 1 |
|  | 1b | Provide an informative summary in the abstract, covering background, objectives, methods, findings/results, interpretation/discussion, and conclusions. | 3-4 |
| **Introduction** | | |  |
| Background | 2 | Provide a background about the rationale of study, what has been previously done, and why this survey is needed. | 5 |
| Purpose/aim | 3 | Identify specific purposes, aims, goals, or objectives of the study. | 5 |
| **Methods** | | |  |
| Study design | 4 | Specify the study design in the methods section with a commonly used term (e.g., cross-sectional or longitudinal). | 6 |
|  | 5a | Describe the questionnaire (e.g., number of sections, number of questions, number and names of instruments used). | 6 |
| Data collection methods | 5b | Describe all questionnaire instruments that were used in the survey to measure particular concepts. Report target population, reported validity and reliability information, scoring/classification procedure, and reference links (if any). | - |
|  | 5c | Provide information on pretesting of the questionnaire, if performed (in the article or in an online supplement). Report the method of pretesting, number of times questionnaire was pre-tested, number and demographics of participants used for pretesting, and the level of similarity of demographics between pre-testing participants and sample population. | 6 |
|  | 5d | Questionnaire if possible, should be fully provided (in the article, or as appendices or as an online supplement). | Appendix s2 |
| Sample characteristics | 6a | Describe the study population (i.e., background, locations, eligibility criteria for participant inclusion in survey, exclusion criteria). | 6 |
|  | 6b | Describe the sampling techniques used (e.g., single stage or multistage sampling, simple random sampling, stratified sampling, cluster sampling, convenience sampling). Specify the locations of sample participants whenever clustered sampling was applied. | 7 |
|  | 6c | Provide information on sample size, along with details of sample size calculation. | - |
|  | 6d | Describe how representative the sample is of the study population (or target population if possible), particularly for population-based surveys. | - |
| Survey  administration | 7a | Provide information on modes of questionnaire administration, including the type and number of contacts, the location where the survey was conducted (e.g., outpatient room or by use of online tools, such as SurveyMonkey). | 6-7, Appendix s3 |
|  | 7b | Provide information of survey’s time frame, such as periods of recruitment, exposure, and follow-up days. | 7 |
|  | 7c | Provide information on the entry process:  –>For non-web-based surveys, provide approaches to minimize human error in data entry.  –>For web-based surveys, provide approaches to prevent “multiple participation” of participants. | -  7 |
| Study preparation | 8 | Describe any preparation process before conducting the survey (e.g., interviewers’ training process, advertising the survey). | - |
| Ethical considerations | 9a | Provide information on ethical approval for the survey if obtained, including informed consent, institutional review board [IRB] approval, Helsinki declaration, and good clinical practice [GCP] declaration (as appropriate). | 6 |
|  | 9b | Provide information about survey anonymity and confidentiality and describe what mechanisms were used to protect unauthorized access. | 6 |
| Statistical  analysis | 10a | Describe statistical methods and analytical approach. Report the statistical software that was used for data analysis. | 7 |
|  | 10b | Report any modification of variables used in the analysis, along with reference (if available). | - |
|  | 10c | Report details about how missing data was handled. Include rate of missing items, missing data mechanism (i.e., missing completely at random [MCAR], missing at random [MAR] or missing not at random [MNAR]) and methods used to deal with missing data (e.g., multiple imputation). | 7 |
|  | 10d | State how non-response error was addressed. | - |
|  | 10e | For longitudinal surveys, state how loss to follow-up was addressed. | - |
|  | 10f | Indicate whether any methods such as weighting of items or propensity scores have been used to adjust for non-representativeness of the sample. | - |
|  | 10g | Describe any sensitivity analysis conducted. | - |
| **Results** | | |  |
| Respondent characteristics | 11a | Report numbers of individuals at each stage of the study. Consider using a flow diagram, if possible. | - |
|  | 11b | Provide reasons for non-participation at each stage, if possible. | - |
|  | 11c | Report response rate, present the definition of response rate or the formula used to calculate response rate. | 8, Table 1 |
|  | 11d | Provide information to define how unique visitors are determined. Report number of unique visitors along with relevant proportions (e.g., view proportion, participation proportion, completion proportion). | - |
| Descriptive  results | 12 | Provide characteristics of study participants, as well as information on potential confounders and assessed outcomes. | 8 |
| Main findings | 13a | Give unadjusted estimates and, if applicable, confounder-adjusted estimates along with 95% confidence intervals and p-values. | - |
|  | 13b | For multivariable analysis, provide information on the model building process, model fit statistics, and model assumptions (as appropriate). | - |
|  | 13c | Provide details about any sensitivity analysis performed. If there are considerable amount of missing data, report sensitivity analyses comparing the results of complete cases with that of the imputed dataset (if possible). | - |
| **Discussion** | | |  |
| Limitations | 14 | Discuss the limitations of the study, considering sources of potential biases and imprecisions, such as non-representativeness of sample, study design, important uncontrolled confounders. | 13 |
| Interpretations | 15 | Give a cautious overall interpretation of results, based on potential biases and imprecisions and suggest areas for future research. | 13 |
| Generalizability | 16 | Discuss the external validity of the results. | 13 |
| **Other sections** | | |  |
| Role of funding source | 17 | State whether any funding organization has had any roles in the survey’s design, implementation, and analysis. | 14 |
| Conflict of interest | 18 | Declare any potential conflict of interest. | 14 |
| Acknowledgements | 19 | Provide names of organizations/persons that are acknowledged along with their contribution to the research. | - |

## Supplement 2: The distributed survey


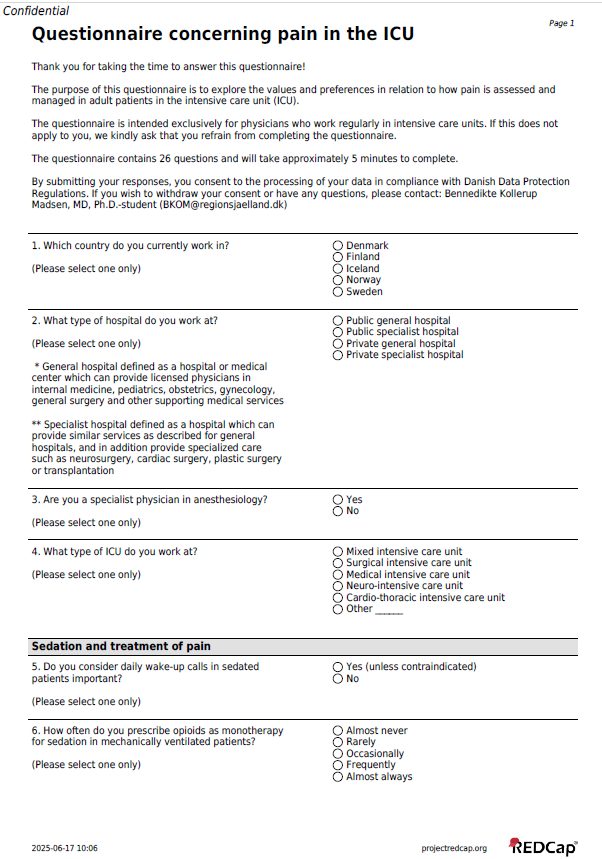


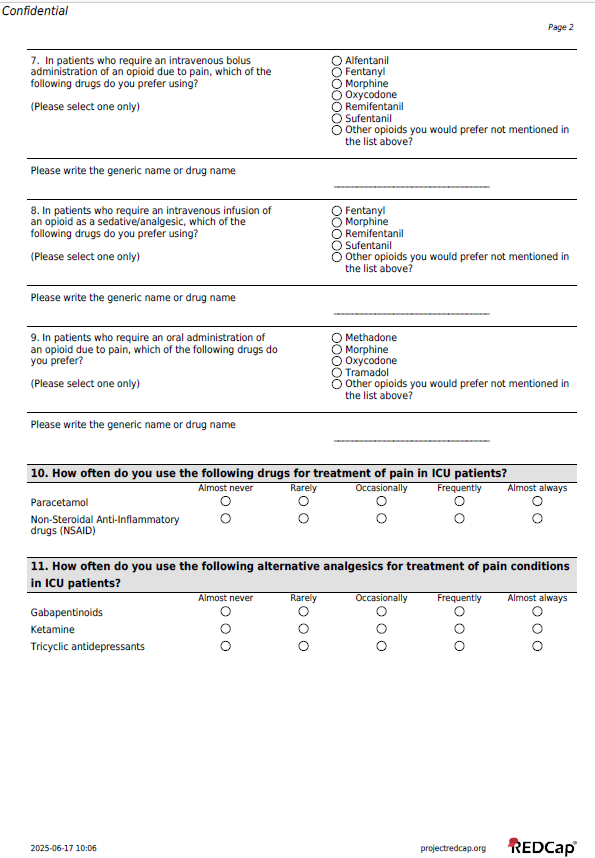


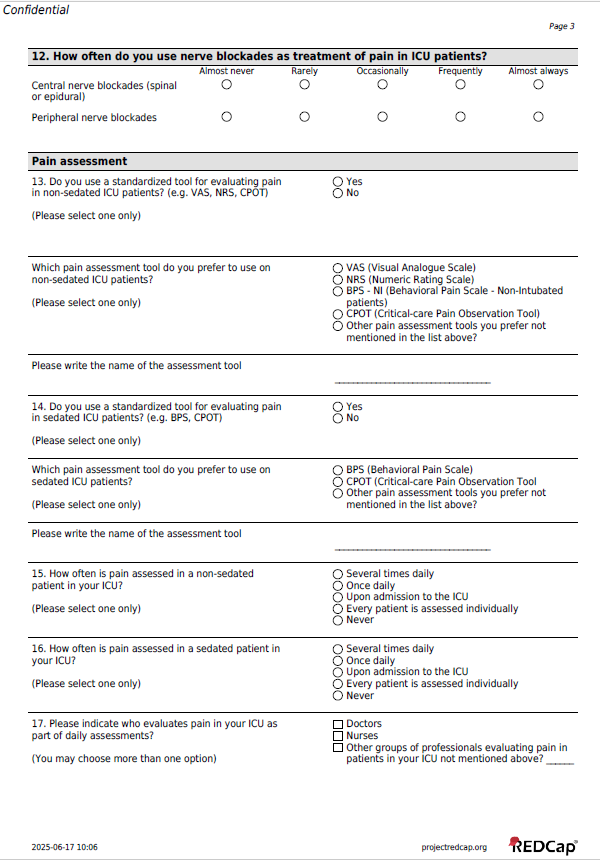


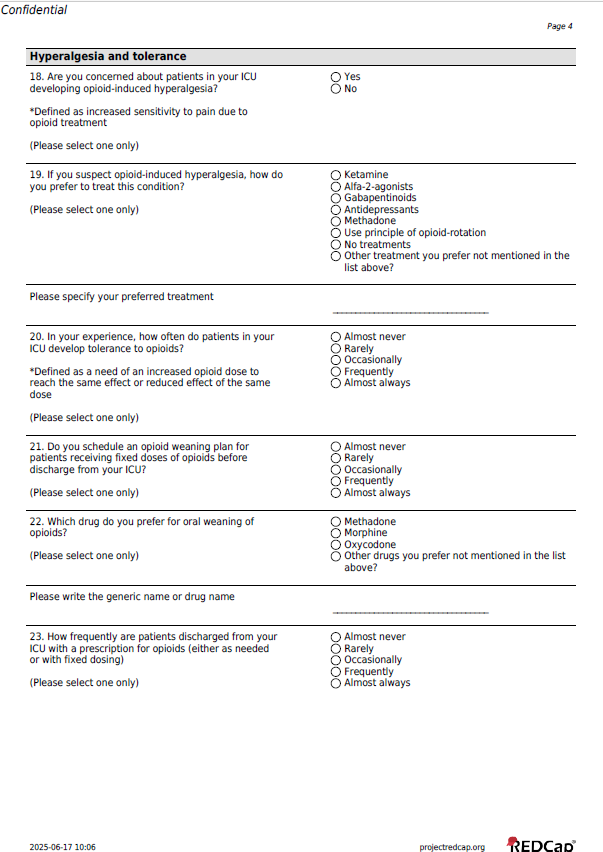


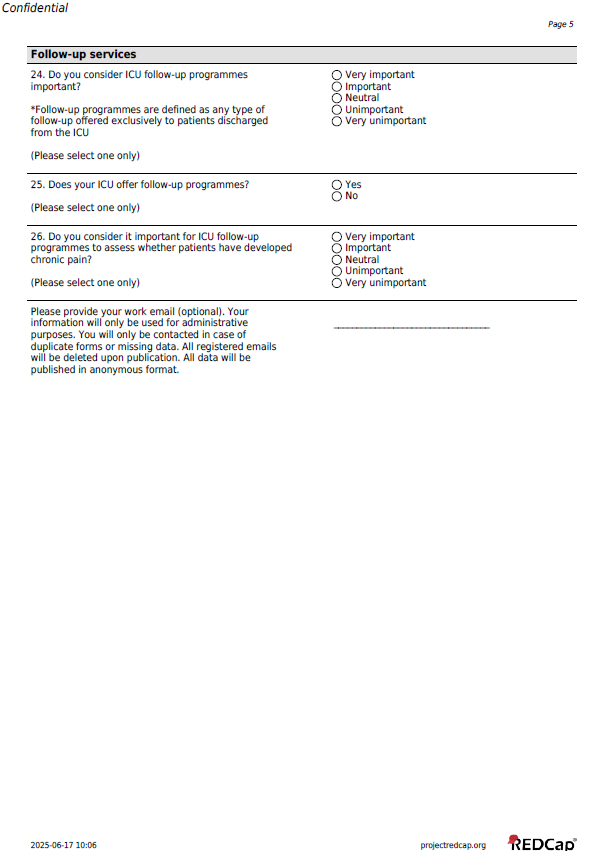


## Supplement 3: Participating countries, sites and investigators

| **Departments/sites** | **Site investigators** |
| --- | --- |
| **Denmark** | |
| Department of Anesthesiology and Intensive Care, Aalborg University Hospital, Aalborg, Denmark | Bodil Steen Rasmussen |
| Section for Neuro and Trauma Intensive Care (NOTIA), Aalborg University Hospital, Aalborg, Denmark | Kirstine Sylvester Conradsen |
| Section for Cardio-thoracic and Vascular Anesthesia and Intensive Care, Aalborg University Hospital, Aalborg, Denmark | Thomas Lass Klitgaard |
| Department of Anesthesiology and Intensive Care, Aarhus University Hospital, Aarhus, Denmark | Asger Granfeldt |
| Department of Anesthesiology and Intensive Care, Copenhagen University Hospital – Bispebjerg Hospital, Copenhagen, Denmark | Theis Skovsgaard Itenov |
| Department of Anesthesiology and Intensive Care, Copenhagen University Hospital – Bornholm Hospital, Bornholm, Denmark | Hans-Christian Thorsen-Meyer |
| Department of Anesthesiology and Intensive Care, Copenhagen University Hospital - Herlev Hospital, Herlev, Denmark | Signe Tellerup Nielsen |
| Department of Anesthesiology and Intensive Care, Copenhagen University Hospital – Hvidovre Hospital, Hvidovre, Denmark | Klaus Tjelle Kristiansen |
| Department of Anesthesiology and Intensive Care, Copenhagen University Hospital -North Zealand Hospital, Hilleroed, Denmark | Morten Heiberg Bestle |
| Department of Intensive Care, Centre for Cancer and Organ Diseases, Copenhagen University Hospital - Rigshospitalet, Copenhagen, Denmark | Stine Estrup |
| Department of Neuroanesthesiology, Copenhagen University Hospital - Rigshospitalet, Copenhagen, Denmark | Kirsten Møller |
| Department of Cardiothoracic Anesthesiology (4141), Copenhagen University Hospital - Rigshospitalet, Copenhagen, Denmark | Peter Hasse Møller Sørensen |
| Department of Anesthesiology and Intensive Care, Goedstrup Hospital, Goedstrup, Denmark | Jeppe Veien Nygaard |
| Department of Anesthesiology and Intensive Care, Holbaek Hospital, Holbaek, Denmark | Kristian Elgaard |
| Department of Anesthesiology and Intensive Care, Lillebaelt Hospital, Vejle, Denmark | Morten Runde Blichfeldt-Eckhardt |
| Department of Anesthesiology and Intensive Care, North Denmark Regional Hospital, Hjoerring, Denmark | Ann Christine Waarkjær Olsen |
| Department of Anesthesiology and Intensive Care, Odense University Hospital, Odense, Denmark | Mette Pedersen |
| Department of Neurosurgical Intensive Care (NIA), Odense University Hospital, Odense, Denmark | Henrik Westy Hoffmeyer |
| Department of Anesthesiology and Intensive Care, Odense University Hospital, Svendborg, Denmark | Peter Martin Hansen |
| Department of Anesthesiology and Intensive Care, Randers Regional Hospital, Randers, Denmark | Helle Bundgaard |
| Department of Anesthesiology and Intensive Care, Slagelse Hospital, Slagelse, Denmark | Susanne Andi Iversen |
| Department of Anesthesiology and Intensive Care, University Hospital of Southern Denmark, Aabenraa Hospital, Aabenraa, Denmark | Marcus Ølgaard Møller |
| Department of Anesthesiology and Intensive Care, University Hospital of Southern Denmark, Esbjerg, Denmark | Jarl Sigaard |
| Department of Anesthesiology and Intensive Care, Zealand University Hospital, Koege, Denmark | Lars Peter Kloster Andersen |
| Department of Anesthesiology and Intensive Care, Zealand University Hospital, Roskilde, Denmark | Lone Musaeus Poulsen |
| Department of Anesthesiology and Intensive Care, Zealand University Hospital, Nykoebing Falster, Denmark | Kim Zillo Rokamp |
| Department of Anesthesiology and Intensive Care, University Hospital of Southern Denmark, Kolding, Denmark | Anne Craveiro Brøchner |
| Department of anesthesiology and Intensive Care, Viborg Regional Hospital, Viborg, Denmark | Christoffer Sølling |
| **Finland** | |
| Intensive Care Unit, Turku University Hospital, Wellbeing Services County of Southwest Finland, Turku, Finland | Mika Valtonen |
| Intensive Care Units, Helsinki University Hospital, Helsinki, Finland | Anna Maria Kuivalainen |
| Intensive Care Unit, Tampere University Hospital, Wellbeing Services County of Pirkanmaa, Tampere, Finland | Annukka Vahtera |
| Intensive Care Unit, Kuopio University Hospital, Wellbeing Services County of North Savo, Kuopio, Finland | Stepani Bendel |
| **Iceland** | |
| Department of Anesthesiology and Intensive Care Medicine, Landspital - The National University Hospital of Iceland, Reykjavik, Iceland | Martin I. Sigurdsson |
| **Norway** | |
| Department of Intensive Care, Akershus Universitetssykehus, Oslo, Norway | Per Martin Bådstøløkken |
| **Sweden** |  |
| Department of Intensive Care and Perioperative Medicine, Skaane University Hospital, Malmö, Sweden | Fredrik Sjövall |

## Supplement 4: Missingness

| **Variable** | **Missing responses** (n=360) | **Missing percentages** |
| --- | --- | --- |
| **Questions 1**  Which country do you currently work in? | 0 | 0.0% |
| **Question 2**  What type of hospital do you work at? | 0 | 0.0% |
| **Question 3**  Are you a specialist physician in anesthesiology? | 0 | 0.0% |
| **Question 4**  What type of ICU do you work at? | 0 | 0.0% |
| **Question 5**  Do you consider daily wake-up calls in sedated patients (unless contraindicated) important? | 1 | 0.3% |
| **Question 6**  How often do you prescribe opioids as monotherapy  for sedation in mechanically ventilated patients? | 0 | 0.0% |
| **Question 7**  In patients who require an intravenous bolus Alfentanil  administration of an opioid due to pain, which of the Fentanyl  following drugs do you prefer using? | 0 | 0.0% |
| **Question 8**  In patients who require an intravenous infusion of Fentanyl  an opioid as a sedative/analgesic, which of the Morphine  following drugs do you prefer using? | 0 | 0.0% |
| **Question 9**  In patients who require an oral administration of Methadone  an opioid due to pain, which of the following drugs do you prefer? | 0 | 0.0% |
| **Question 10**  How often do you use the following drugs for treatment of pain in ICU patients? | 0 | 0.0% |
| **Question 11**  How often do you use the following alternative analgesics for treatment of pain conditions  in ICU patients? | 0 | 0.0% |
| **Question 12**  How often do you use nerve blockades as treatment of pain in ICU patients? | 0 | 0.0% |
| **Question 13**  Do you use a standardized tool for evaluating pain  in non-sedated ICU patients? (e.g. VAS, NRS, CPOT) | 0 | 0.0% |
| **Question 14**  Do you use a standardized tool for evaluating pain  in sedated ICU patients? (e.g. BPS, CPOT) | 0 | 0.0% |
| **Question 15**  How often is pain assessed in a non-sedated  patient in your ICU? | 0 | 0.0% |
| **Question 16**  How often is pain assessed in a sedated patient in your ICU? | 1 | 0.3% |
| **Question 17**  Please indicate who evaluates pain in your ICU as part of daily assessments? | 0 | 0.0% |
| **Question 18**  Are you concerned about patients in your ICU developing opioid-induced hyperalgesia? | 0 | 0.0% |
| **Question 19**  If you suspect opioid-induced hyperalgesia, how do you prefer to treat this condition? | 0 | 0.0% |
| **Question 20**  In your experience, how often do patients in your ICU develop tolerance to opioids? | 0 | 0.0% |
| **Question 21**  Do you schedule an opioid weaning plan for patients receiving fixed doses of opioids before discharge from your ICU? | 0 | 0.0% |
| **Question 22**  Which drug do you prefer for oral weaning of opioids? | 0 | 0.0% |
| **Question 23**  How frequently are patients discharged from your ICU with a prescription for opioids (either as needed or with fixed dosing) | 0 | 0.0% |
| **Question 24**  Do you consider ICU follow-up programs important? | 0 | 0.0% |
| **Question 25**  Does your ICU offer follow-up programmes? | 0 | 0.0% |
| **Question 26**  Do you consider it important for ICU follow-up programmes to assess whether patients have developed chronic pain? | 0 | 0.0% |

## Supplement 5: Pain assessment tools

**Figure S1: Preferred pain assessment tool in the ICU – non-sedated patients**.

**
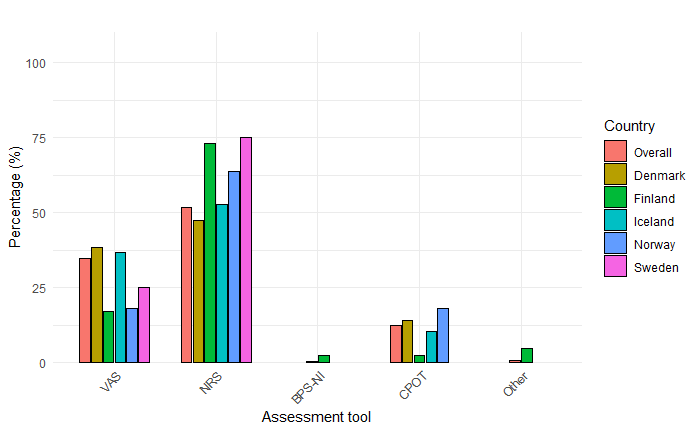
**

*VAS: Visual Analogue Scale, NRS: Numerical Rating Scale, BPS-NI: Behavioral Pain Scale Non-intubated, CPOT: Critical Care Pain Observation Tool*.

**Figure S2: Preferred pain assessment tool in the ICU – sedated patients.**

**
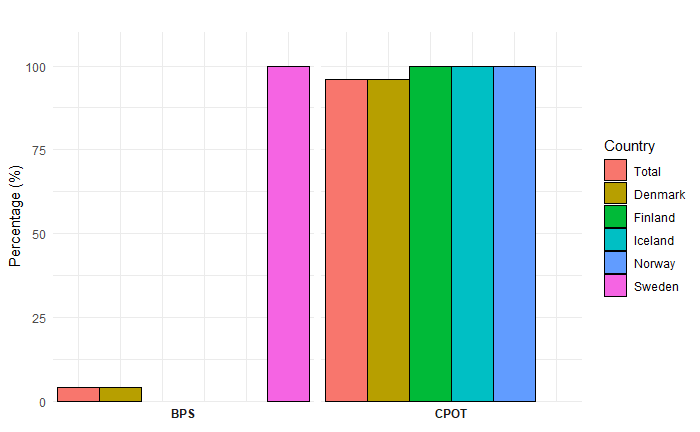
**

*BPS: Behavioral Pain Scale, CPOT: Critical Care Pain Observation Tool*

## Supplement 6: Pain assessment frequency

**Figure S3: Frequency of pain assessments in ICU patients – non-sedated patients.**

**
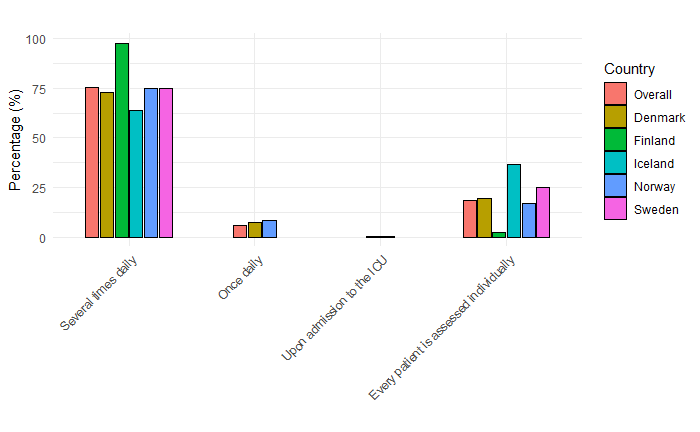
**

**Figure S4: Frequency of pain assessments in ICU patients – sedated patients.**

*
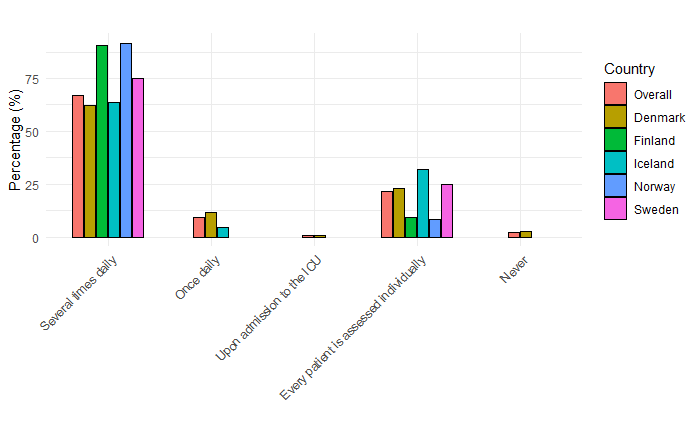
*

## Supplement 7: Wake-up calls

**Figure S5: “Do you consider daily wake-up calls in sedated patients important?”**


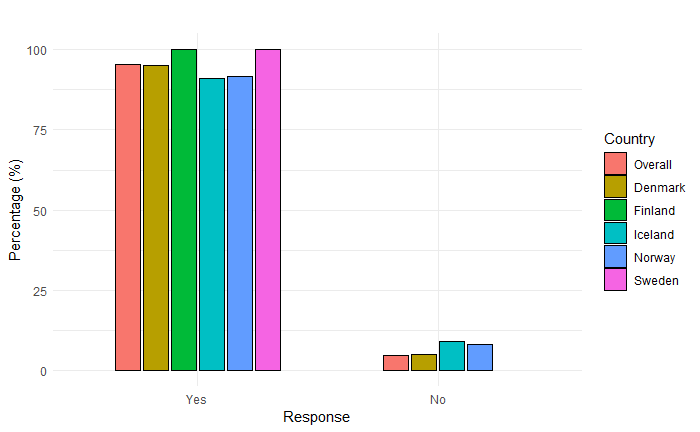


## Supplement 8: Opioids as monotherapy for sedation

**Figure S6: Opioids as monotherapy for sedation in mechanically ventilated ICU patients.**


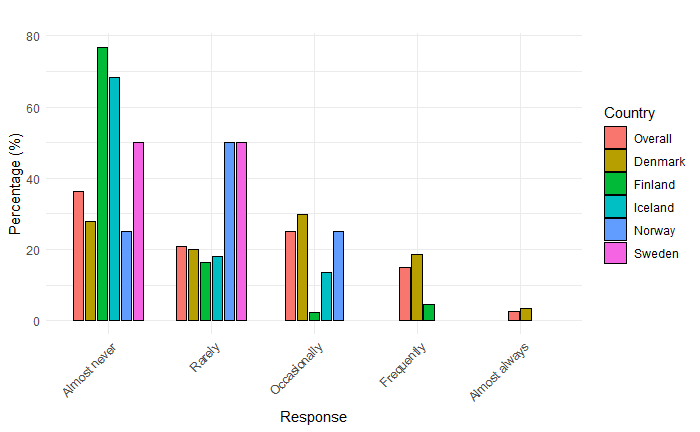


## Supplement 9: Non-opioid analgesics

**Figure S7: Use of paracetamol for treatment of pain in ICU patients.**


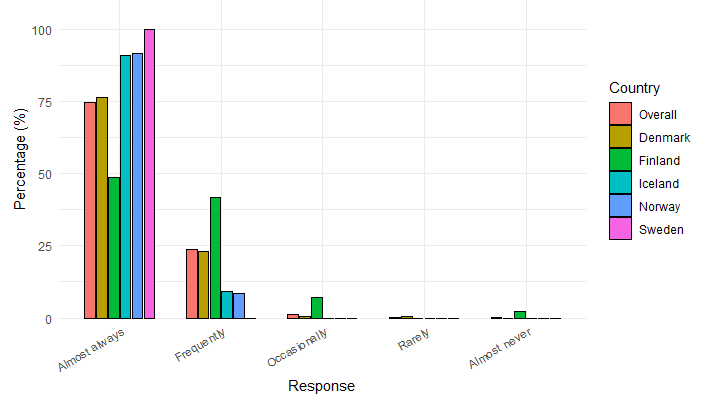


**Figure S8: Use of non-steroidal anti-inflammatory drugs for treatment of pain in ICU patients.**


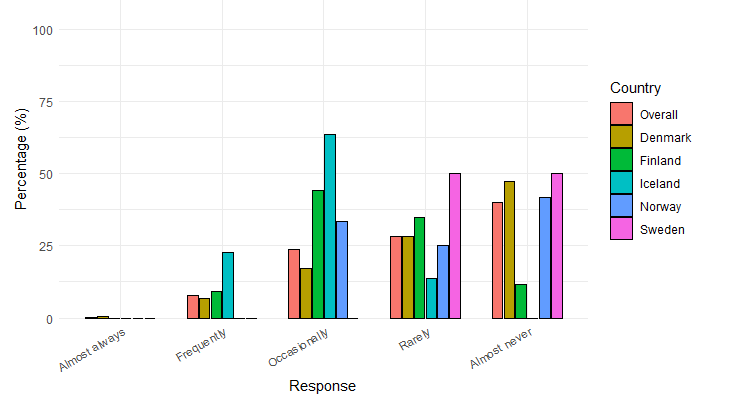


**Figure S9: Use of gabapentinoids for treatment of pain in ICU patients.**

**
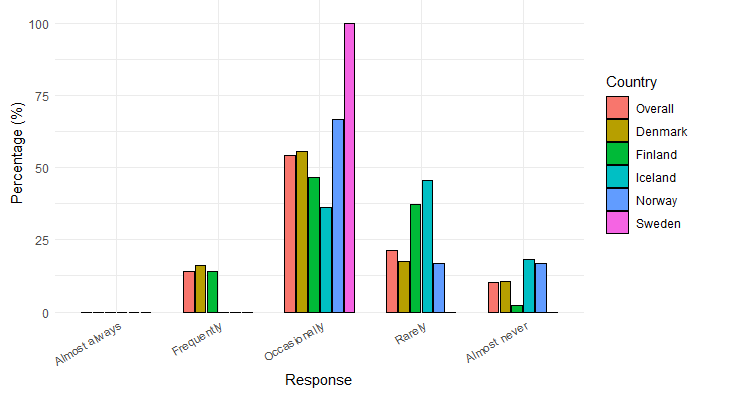
**

**Figure S10: Use of ketamine for treatment of pain in ICU patients.**

**
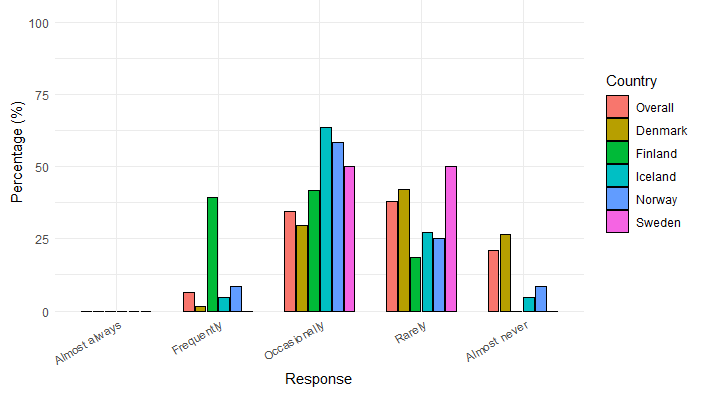
**

**Figure S11: Use of tricyclic antidepressants for treatment of pain in ICU patients.**

**
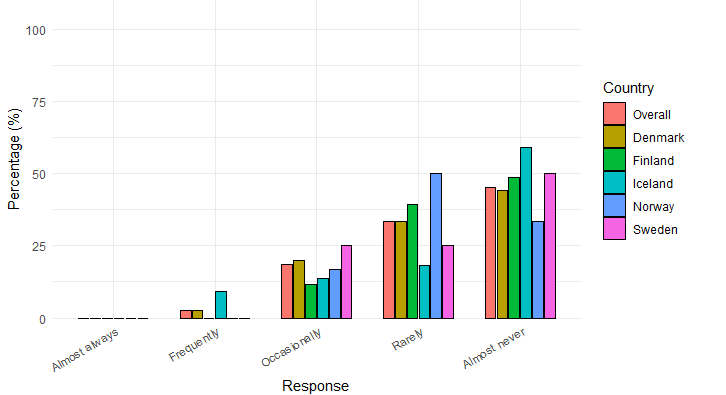
**

**Figure S12: Use of central nerve blockades for treatment of pain in ICU patients.**

**
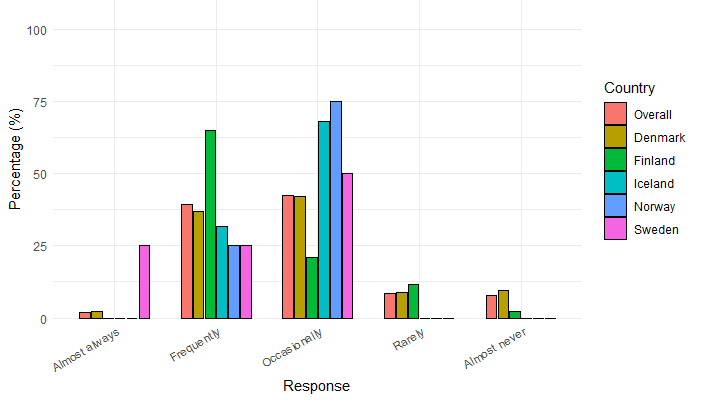
**

**Figure S13: Use of peripheral nerve blockades for treatment of pain in ICU patients.**

**
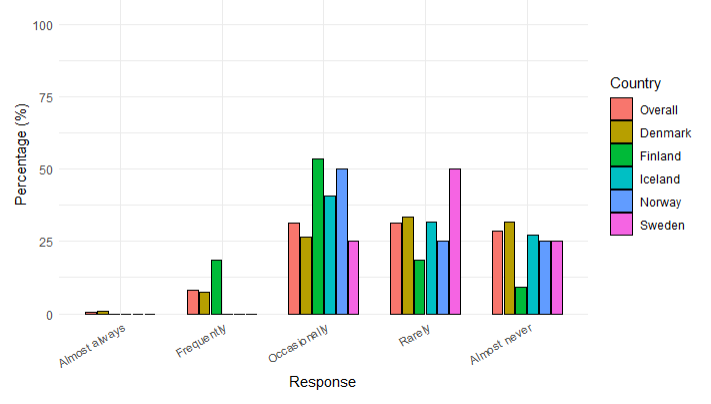
**

## Supplement 10: Opioid-induced hyperalgesia

**Figure S14: Concerns about opioid-induced hyperalgesia in ICU patients**.


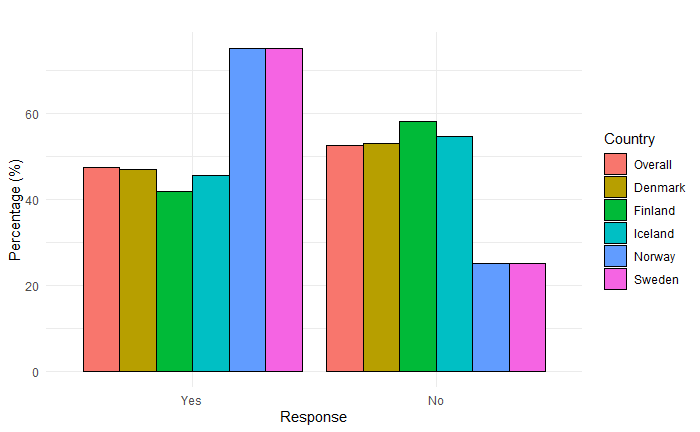


**Figure S15: Preferred treatments if opioid-induced hyperalgesia is suspected in ICU patients.**


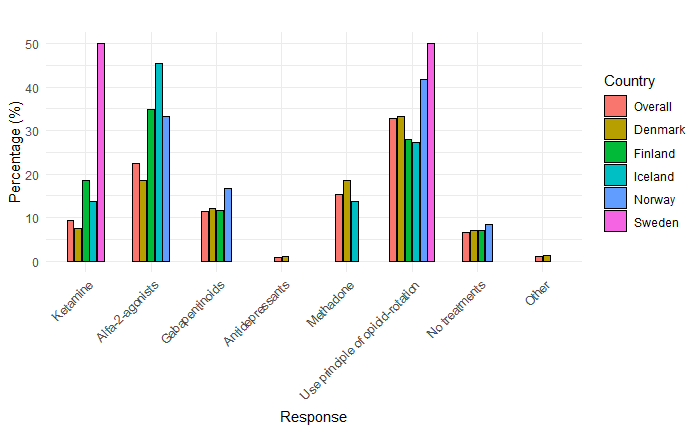


## Supplement 11: Opioid tolerance

**Figure S16: Physicians’ experiences regarding development of opioid tolerance in ICU patients.**

**
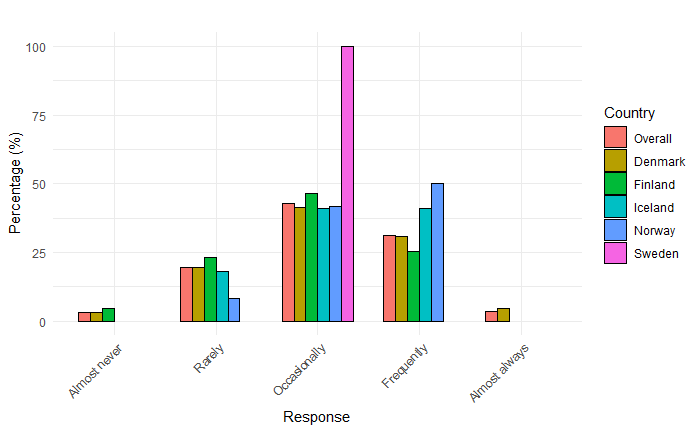
**

## Supplement 12: Opioid weaning

**Figure S17: Opioid weaning plan before ICU discharge.**


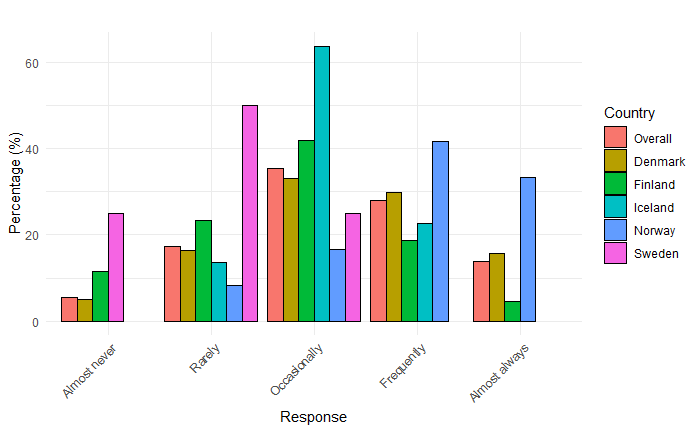


**Figure S18: Preferred drug treatment for oral opioid weaning in ICU patients**


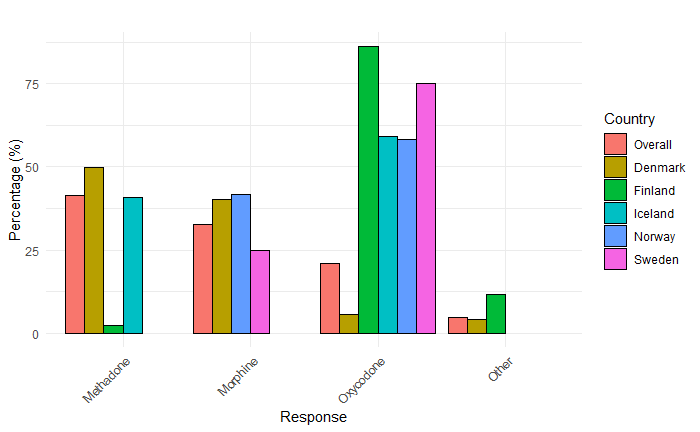


*Among the 17 respondents (4.7%) of respondents who selected the “other” option, 8 respondents (47%) mentioned clonidine, 5 respondents (29%) mentioned buprenorphine. The remaining 4 respondents ( 24%) did not write a specific drug*.

##

## Supplement 13: Opioid prescription

**Figure S19: Frequency of ICU patients being discharged with prescriptions to opioids**.

**
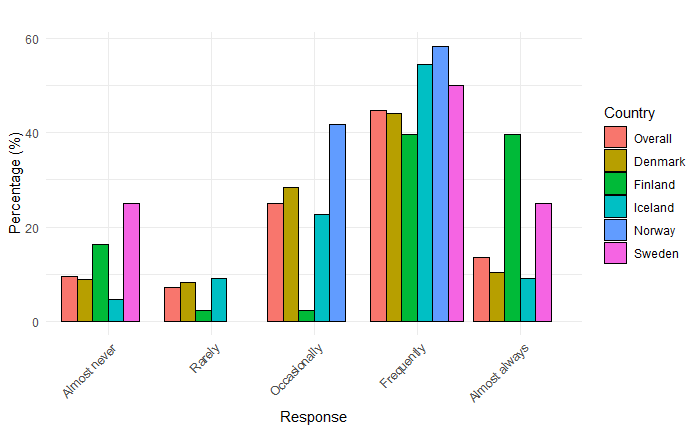
**

## Supplement 14: ICU follow-up programs

**Figure S20: Perceived importance of ICU follow-up programs.**


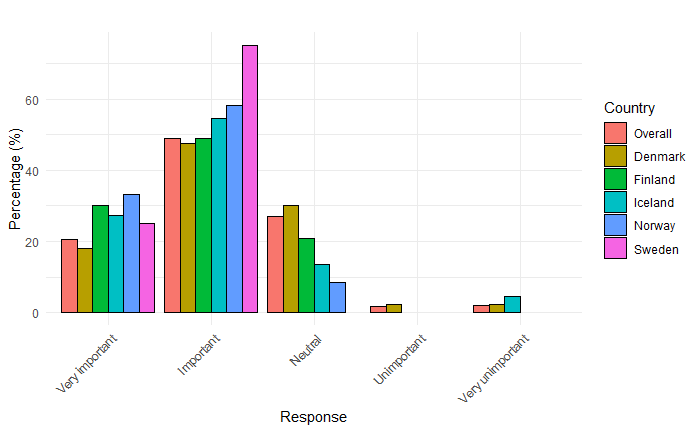


**Figure S21: Availability of ICU follow-up programs.**

**
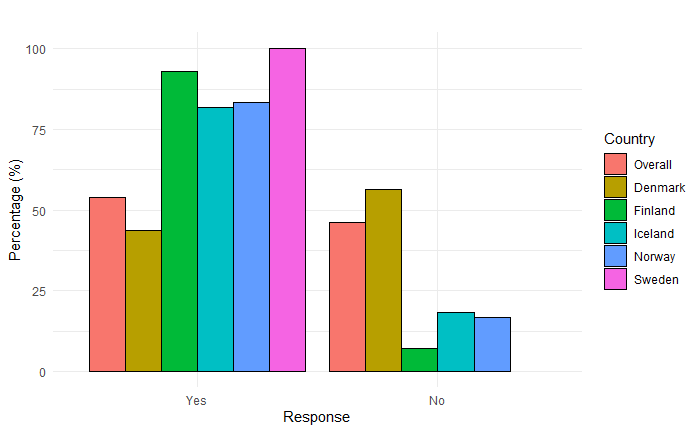
**

**Figure S22: Perceived importance of assessing chronic pain in ICU follow-up programs.**

**
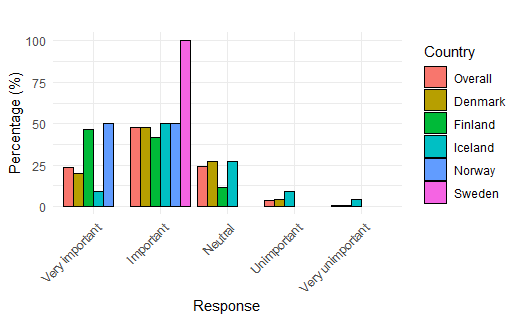
**
